# Editorial: Model organisms in neuropharmacology 2024

**DOI:** 10.3389/fphar.2026.1800010

**Published:** 2026-02-13

**Authors:** Veronica Rivi, Attila Sik, Cristina Benatti, Alejandra M. Pacchioni

**Affiliations:** 1 Department of Life Sciences, University of Modena and Reggio Emilia, Modena, Italy; 2 Institute of Physiology, Medical School, University of Pecs, Pecs, Hungary; 3 Obuda University, John von Neumann Faculty of Informatics, Biomatics and Applied Artificial Intelligence Institute, Budapest, Hungary; 4 Department of Biomedical, Metabolic, and Neural Sciences, University of Modena and Reggio Emilia, Modena, Italy; 5 Toxicology Area, School of Biochemical and Pharmaceutical Sciences, National University of Rosario (UNR), National Scientific Council (CONICET), Rosario, Argentina

**Keywords:** face validity, construct validity, predictive validity, cross-species integration, preclinical models, rodent models, zebrafish, *Lymnaea stagnalis*

Model organisms have long served as–and still represent - the conceptual and experimental cornerstone for scientific research (Wilson-Sanders, 2011; Rivi et al., 2023). From target discovery to behavioral phenotyping, they enable the characterization of neural and molecular processes that would otherwise remain inaccessible. Yet the same systems that have driven foundational advances also define many of the field’s current limitations. Despite transformative progress in genetics, imaging, and systems-level neuroscience, translating preclinical findings into effective and safe therapies for brain disorders remains limited (Seyhan, 2019). While neurodegenerative and neuropsychiatric diseases continue to impose a substantial global burden (Hay et al., 2025), prolonged timelines and escalating costs of preclinical studies, as well as high rates of clinical failure, delay central nervous system drug development. These challenges reflect not only the intrinsic complexity of the brain but also the selection, validation, and integration of experimental models across the preclinical pipeline.

As neuropsychiatric disorders emerge from dynamic interactions among genetic susceptibility, biological sex, early-life development, environmental factors, stress, and aging, no single experimental model can capture this multidimensional complexity in full. At the same time, increasing pressure to reduce animal use and concern over the environmental persistence of neuroactive compounds demand approaches that are both scientifically rigorous and ethically responsible. Consequently, a shift away from model-centric thinking toward strategies that explicitly align specific organisms with clearly defined experimental goals is both necessary and timely.

**FIGURE 1:**
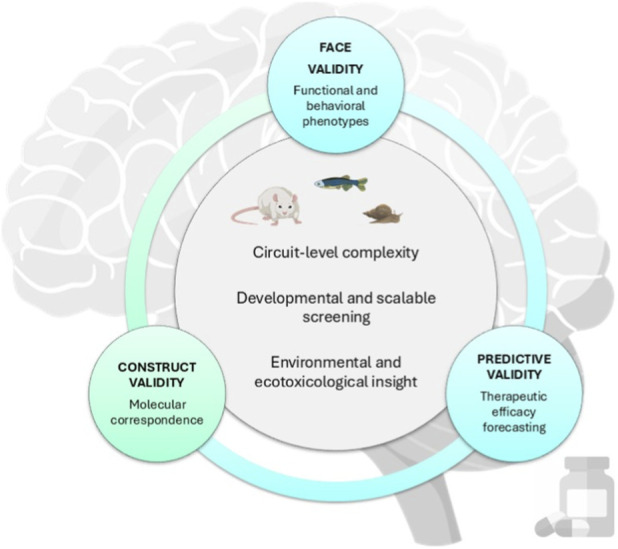
Integrative use of model organisms across validity domains in neuropharmacology.

*Model Organisms in Neuropharmacology* was designed to illustrate how mammalian and non-mammalian systems can be used in a complementary way to enhance understanding, ensure translational relevance, and promote responsible research practices. Across the Research Topic, model organisms are presented as interconnected systems that enable the investigation of different neuropharmacological processes at molecular, cellular, behavioral, and organismal levels. Central to this approach is the consideration of experimental validity. *Face validity* reflects phenotypic similarity to human disease, *construct validity* denotes correspondence with underlying pathophysiology, and *predictive validity* captures the ability of a model to forecast therapeutic efficacy. Although these dimensions of validity are analytically distinct, they often overlap in practice and are most informative when considered together. However, because no single model can fully recapitulate all three dimensions, cross-species integration is required (Figure 1). In this Research Topic, model organisms are strategically adopted to address distinct dimensions of experimental validity and to refine hypotheses across molecular, circuit, and behavioral levels. This framework provides the context for the individual contributions that follow, each of which addresses a specific translational bottleneck in neuropharmacology.

One such bottleneck is the limited translation of molecular and circuit-level findings into meaningful functional and cognitive outcomes. This challenge is addressed in the systematic review by Foo et al., which synthesizes evidence from rodent models of genetic epilepsy and demonstrates that neurocognitive and affective impairments are common, yet highly dependent on genotype and developmental stage. By extending analysis beyond seizure-centric phenotypes, this work strengthens face validity and underscores the importance of incorporating behavioral and cognitive endpoints when evaluating disease mechanisms and therapeutic efficacy. Notably, the genotype- and development-dependent variability highlighted in this review also emphasizes the value of scalable screening platforms, such as larval zebrafish seizure paradigms, for early-stage compound prioritization across developmental windows prior to targeted validation in mammalian systems.

Construct validity is exemplified by Zhukov et al., who investigate trace amine-associated receptor 9 (TAAR9) knockout rats. Their findings link targeted genetic disruption to alterations in hippocampal serotonin levels and grooming behavior, providing a mechanistic bridge between molecular signaling pathways and affective or compulsive-like phenotypes relevant to psychiatric disorders. Such genetically informed mammalian models support hypothesis-driven pharmacological interrogation within well-defined neurobiological frameworks. Several contributions further emphasize that pharmacological efficacy often emerges from coordinated, system-level modulation rather than isolated molecular targets. In a rodent model of ischemic stroke, Athirah et al. demonstrate that a combination of medicinal mushroom extracts produces neuroprotective effects extending beyond lesion size reduction to include improvements in cognitive and motor function, thereby enhancing both face and predictive validity. Similarly, Lan et al. show that tetramethylpyrazine preserves neural integrity and cognitive performance in a hypobaric hypoxia model, highlighting the translational relevance of *in vivo* neuroprotective strategies that engage metabolic, electrophysiological, and cognitive domains within intact organisms.

Developmental and environmental neuropharmacology represent another key focus of the Research Topic. Jensen et al. review evidence that developmental exposure to the endocrine-disrupting chemicals bisphenol A and tributyltin disrupts estrogen signaling and induces long-lasting functional deficits in the zebrafish retina. Zebrafish offer a favorable balance of construct validity, through conserved molecular pathways, and face validity, through direct assessment of functional neural outcomes following early-life perturbations. This work underscores the importance of developmental timing in shaping neuropharmacological vulnerability.

Zebrafish models also contribute to predictive validity by enabling rapid and scalable assessment of neuroactive compounds. Schneider et al. demonstrate how larval zebrafish seizure paradigms can be used to evaluate the anticonvulsant potential of the flavonoid luteolin, combining behavioral screening with mechanistic inference. Such approaches facilitate efficient compound prioritization, reduce experimental burden, and support subsequent validation in mammalian systems.

The inclusion of invertebrate systems extends neuropharmacological investigation to organism-wide and ecological contexts. Ilyaskina et al. use the pond snail *Lymnaea stagnalis* to assess the effects of chronic fluoxetine exposure across adult and developmental stages, integrating lipidomic, metabolomic, and classical endpoints. While such systems may have limited face validity for complex human neuropsychiatric disorders, their value lies in construct-level insight, predictive screening efficiency, and the ability to reveal systemic and developmental consequences of neuroactive pharmaceuticals that are difficult to isolate in mammalian models. Complementing this perspective, Pan et al. demonstrate that Jiannao Pills alleviate depression-like behavior in a chronic stress mouse model via modulation of the NF-κB/NLRP3 inflammatory pathway, reinforcing the relevance of multi-component pharmacological interventions and positioning neuroimmune signaling as a key axis for predictive validity in affective disorder models.

Thus, by aligning experimental models with explicit validity goals, this Research Topic demonstrates that organisms are most informative when strategically integrated within a framework that balances face, construct, and predictive validity, thereby defining a rigorous and scalable path toward neuropharmacological research that is more efficient, mechanistically precise, and genuinely translational.

No single experimental system satisfies all dimensions of experimental validity. Face validity is strengthened through functional and behavioral outcomes, construct validity through mechanistic and molecular correspondence, and predictive validity through alignment with clinically meaningful endpoints. Comparative integration of mammalian and non-mammalian models enables complementary strengths across these domains, supporting more predictive, efficient, and responsible neuropharmacological research.
